# Crystal structure of tyrosine decarboxylase and identification of key residues involved in conformational swing and substrate binding

**DOI:** 10.1038/srep27779

**Published:** 2016-06-13

**Authors:** Haixia Zhu, Guochao Xu, Kai Zhang, Xudong Kong, Ruizhi Han, Jiahai Zhou, Ye Ni

**Affiliations:** 1 The Key Laboratory of Industrial Biotechnology, Ministry of Education, School of Biotechnology, Jiangnan University, Wuxi 214122, Jiangsu, People’s Republic of China; 2 Shanghai Institute of Organic Chemistry, State Key Laboratory of Bioorganic & Natural Products Chemistry, Chinese Academy of Sciences, Shanghai 200032, People’s Republic of China; 3 State Key Laboratory of Bioreactor Engineering, School of Biotechnology, East China University of Science and Technology, Shanghai 200237, People’s Republic of China

## Abstract

Tyrosine decarboxylase (TDC) is a pyridoxal 5-phosphate (PLP)-dependent enzyme and is mainly responsible for the synthesis of tyramine, an important biogenic amine. In this study, the crystal structures of the apo and holo forms of *Lactobacillus brevis* TDC (*Lb*TDC) were determined. The *Lb*TDC displays only 25% sequence identity with the only reported TDC structure. Site-directed mutagenesis of the conformationally flexible sites and catalytic center was performed to investigate the potential catalytic mechanism. It was found that H241 in the active site plays an important role in PLP binding because it has different conformations in the apo and holo structures of *Lb*TDC. After binding to PLP, H241 rotated to the position adjacent to the PLP pyridine ring. Alanine scanning mutagenesis revealed several crucial regions that determine the substrate specificity and catalytic activity. Among the mutants, the S586A variant displayed increased catalytic efficiency and substrate affinity, which is attributed to decreased steric hindrance and increased hydrophobicity, as verified by the saturation mutagenesis at S586. Our results provide structural information about the residues important for the protein engineering of TDC to improve catalytic efficiency in the green manufacturing of tyramine.

Biogenic amines (BAs) are essential, biologically important nitrogenous compounds that play important roles in intercellular communication as major transmitters^1^. Many BAs, including dopamine, serotonin, histamine and tyramine, generally exist in microbial, plant and animal cells^2^. Tyramine is a naturally occurring BA and has diverse applications. It could act as an endogenous ligand for mammalian trace amine-associated receptors and as a biochemical precursor for dopamine and octopamine, which are neurotransmitters and neuromodulators in invertebrate nervous systems^3^,^4^,^5^,^6^. Tyramine has also been shown to be an effective therapeutic agent in Diabetes Mellitus type II by functioning as an insulin mimic to stimulate glucose transport^7^, and as a key intermediate of Bezafibrate for the treatment of hyperlipidemia by reducing cholesterol and triglyceride levels^8^. Similar to dopamine, the presence of higher concentrations of tyramine *in vivo* could be regarded as an indicator of health problems, such as cardiovascular arrest/stroke. Although the industrial production of tyramine has been limited to chemical processes, enzymatic decarboxylation of tyrosine by a decarboxylase is much more promising, due to its green properties and high efficiency.

Amino acid decarboxylases are PLP-dependent enzymes that are responsible for the biosynthesis of BAs and polyamines, and can be divided into four evolutionally distant subgroups based on analyses of the primary sequence alignments and the conserved domains^9^. Tyrosine decarboxylase (TDC, EC 4.1.1.25) is classified into Group II and shares high identity with L-3,4-dihydroxy-L-phenylalanine (DOPA) decarboxylase (DDC), glutamic acid decarboxylase (GAD), and histidine decarboxylase (HDC). TDC could catalyze the decarboxylation of L-tyrosine and L-DOPA to tyramine and dopamine^10^,^11^. To date, various TDCs have been identified from different organisms, such as plants^12^,^13^, insects^14^,^15^, and different microorganisms including *Lactobacillus brevis*^10^,^16^, *Enterococcus faecalis*^17^, *Methanocaldococcus jannaschii*^18^, *Sporolactobacillus* sp.^19^, and *Lactococcus lactis*^20^. Previous studies show that lactic acid bacteria (LAB) are one major class of TDC producers. From an evolutionary point of view, the TDC system in LAB is responsible for tyramine formation in response to acid challenge. The TDC system could generate a proton motive force (PMF) through proton consumption in the decarboxylation reaction and the membrane potential resulting from electrogenic transport of tyrosine in exchange for its corresponding biogenic amine tyramine^21^. However, despite its important applications, only one TDC structure from *Methanococcus jannaschii* (PDB code: 3F9T, *Mj*TDC) has been resolved and deposited in the PDB database, and the key residues responsible for substrate binding and the catalytic mechanism remain unclear.

In our previous study, we identified a TDC coding gene from *Lactobacillus brevis* CGMCC 1.2028 and successfully expressed the soluble protein in *Escherichia coli* BL21(DE3)^10^. A BLASTp search based on the amino acid sequence of *Lactobacillus brevis* TDC (*Lb*TDC) reveals that *Lb*TDC shares only 25% sequence identity and 60% coverage with *Mj*TDC. Importantly, *Lb*TDC is superior to *Mj*TDC due to its lower *K*_m_ (0.58 versus 1.6 mM) and higher specific activity (43.1 versus 1.1 U·mg^−1^)^18^. To better understand its catalytic mechanism, the *Lb*TDC protein was crystallized and the X-ray diffraction patterns were collected in this study. Furthermore, structure-guided site-directed mutagenesis and alanine scanning and saturation mutagenesis of *Lb*TDC were performed on residues around the substrate binding sites and those required for conformational stability to elucidate the function of key residues involved in the catalytic mechanism and to promote the potential applications of *Lb*TDC in tyramine synthesis, food safety, and pharmacology.

## Materials and Methods

### Chemicals, reagents, strains and plasmids

Tyrosine, PLP, tyramine, DOPA, dopamine, SeMet and all reagents for protein crystallization were purchased from Sigma-Aldrich Co., Ltd. (St. Louis, MO, USA). *E. coli* BL21(DE3) harboring pET24a-*tdc* was constructed in our previous work^10^. Phanta HS Super-Fidelity DNA Polymerase was obtained from Vazyme Biotech Co., Ltd. (Nanjing, China), and *Dpn*I was obtained from Toyobo Co., Ltd. (Osaka, Japan).

### Protein expression and purification

Recombinant *E. coli* BL21(DE3) harboring pET24a-*tdc* was cultivated at 37 °C and 180 rpm in a LB medium supplemented with 1% glucose and 50 μg mL^–1^ kanamycin until the OD_600_ reached 0.6–0.8; then, 0.2 mM isopropyl-β-D-thiogalactopyraniside (IPTG) was added, and the bacteria were further cultured at 16 °C. After induction for 16 h, the cells were harvested by centrifugation (8,000×*g* for 10 min), resuspended in buffer A (25 mM Tris-HCl, 300 mM NaCl, 20 mM imidazole, 5 mM β-mercaptoethanol, pH 7.4, and 1 mM phenyl-methyl-sulfonyl fluoride), and then disrupted using a high-pressure homogenizer (ATS BASIC-II, Canada). The cell lysate was centrifuged (15,000×*g* for 30 min) at 4 °C to remove the cell debris, and the resulting supernatant was loaded onto a Fast Flow Ni^2+^-agarose column (GE Healthcare, USA), which was pre-equilibrated with buffer A. Afterwards, the retained protein was eluted with a 20–300 mM imidazole gradient in buffer B (25 mM Tris-HCl, 300 mM NaCl, 300 mM imidazole, and 5 mM β-mercaptoethanol, pH 7.4). The eluents were concentrated, desalted, and stored in buffer containing 25 mM Tris-HCl, 150 mM NaCl, and 1 mM DL-Dithiothreitol, pH 7.4. After the eluents were concentrated to approximately 10 mg·mL^−1^, the purified apo-*Lb*TDC protein was immediately frozen in liquid nitrogen and stored at −80 °C for crystallization. The molecular weight was analyzed by SDS-PAGE and Superdex-200 gel filtration (GE Healthcare, USA). The selenomethionine-substituted *Lb*TDC (SeMet-*Lb*TDC) protein was prepared using previously reported methods^22^ and purified as described above.

### Crystallization

Initial crystallization was performed at 18 °C using the sitting-drop vapor diffusion method and commercial crystallization kits. Based on the obtained preliminary crystallization condition, the pH of the buffer and concentration of the precipitant were further optimized to obtain regular crystals. The best crystals were observed in the droplets obtained by mixing 2 μL of protein solution (10 mg·mL^−1^) with 2 μL of reservoir solution, which consisted of 0.1 M sodium cacodylate trihydrate (pH 7.5), 0.2 M MgCl_2_, and 18% PEG 1000. The SeMet-*Lb*TDC crystals were obtained in 0.1 M sodium cacodylate trihydrate, pH 7.0, 0.1 M MgCl_2_, and 16% PEG 4000. The *Lb*TDC/PLP complex was also crystallized at a molar ratio of 1:4 under the same conditions as SeMet-*Lb*TDC. Crystals were usually obtained in 2 to 5 days.

### Data collection, structure determination and refinement

For X-ray diffraction, all crystals were equilibrated in a cryoprotectant solution containing 90% reservoir solution and 10% glycerol and then flash-cooled with liquid nitrogen. Diffraction data for SeMet-*Lb*TDC were collected using beamline BL17A at the High Energy Accelerator Research Organization (KEK, Japan). The data for the native protein and *Lb*TDC-PLP complex were collected using beamlines BL17U1 and BL19U1 at the Shanghai Synchrotron Radiation Facility (SSRF, China), respectively. All of the diffraction images were integrated and scaled with the HKL2000 package^23^. The crystal parameters and data collection statistics are listed in Table 1.

The phases of *Lb*TDC were solved using the Multi-wavelength anomalous diffraction method. The position of the Se atom was identified by Autosol^24^. Then, AutoBuild^25^ in the Phenix package was employed to automatically build the model of *Lb*TDC. Several cycles of refinement were performed using Phenix^26^ and Coot^27^. The structure of the *Lb*TDC/PLP complex was solved by the molecular replacement method using Phaser^28^ in the CCP4 crystallographic suite^29^, and the crystal structure of *Lb*TDC was used as a search model.

### Mutagenesis

Site-directed mutagenesis of *Lb*TDC was performed by PCR using pET24a-*tdc* as a template and Phanta HS Super-Fidelity DNA Polymerase. The resulting PCR products (10 μL) were digested with 0.2 μL of *Dpn*I for 2 h at 37 °C to remove the methylated templates. Then, the whole plasmids were transformed into *E. coli* JM109 and cultivated on LB agar plates containing 50 μg·mL^−1^ kanamycin. The mutated plasmids were confirmed by sequencing and further transformed into *E. coli* BL21 (DE3) for over-expression. The primers used in this study are shown in Table S1.

### Enzymatic activity and kinetic assay

The enzymatic activity of *Lb*TDC was determined using a previously reported method^10^, with some modifications. The assay mixture (1 mL) consisted of 2.75 mM L-tyrosine or L-DOPA in sodium acetate buffer (0.2 M, pH 5.0), 0.2 mM PLP, and an appropriate amount of enzyme in buffer (25 mM Tris-HCl and 150 mM NaCl, pH 7.4). The reaction was incubated at 40 °C for 10 min and terminated by boiling at 100 °C for 10 min. The tyrosine, tyramine, DOPA, and dopamine levels were quantified by HPLC (Agilent 1260, USA) equipped with a Diamonsil C18 column (DIKMA, China) using water/methanol (90/10) as eluent at 1.0 mL·min^−1^, 220 nm, and 30 °C. One unit of activity was defined as the amount of enzyme that was required for the production of 1.0 μmol tyramine or dopamine per minute under the above conditions. The kinetic parameters were determined in the presence of varying concentrations of tyrosine and DOPA from 0.1 to 5.5 mM and calculated by non-linear curve fitting using Origin8.0. All assays were performed in triplicate.

### Molecular docking

All docking calculations were accomplished with AutoDock Vina 1.1. A docking algorithm that takes into account the ligand flexibility but keeps the protein rigid was employed. Docking runs were performed using the standard parameters of the program for interactive growing and subsequent scoring. However, the parameters used to set the grid box dimensions and center were: center_x = 33.121; center_y = −7.691; center_z = 77.977; size_x = 15; size_y = 15; size_z = 15.

## Results and Discussion

### Overall structures of *Lb*TDC

After optimization of the crystallization conditions, the crystals of *Lb*TDC alone and in complex with PLP (apo-*Lb*TDC and holo-*Lb*TDC, respectively) were obtained and the corresponding three-dimensional structures were resolved. The *Lb*TDC crystals were diffracted at 1.90 Å and belonged to the space group P2_1_. The final structure was refined to R_work_ of 17.13% and R_free_ of 21.28%. The crystallographic statistics for data collection and refinement are listed in Table 1. The structures of *Lb*TDC alone and in complex with PLP have been deposited in the PDB database under accession Nos. 5HSI and 5HSJ.

**Figure 1:**
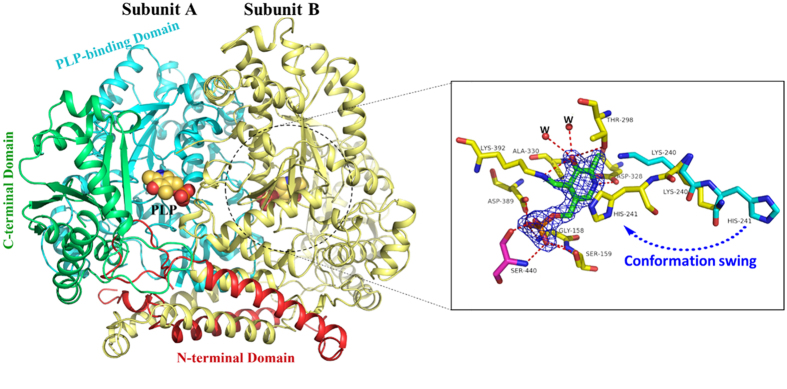
Overall crystal structure of *Lb*TDC in complex with PLP. The *N*-terminal domain (red), PLP-binding (cyan) and C-terminal (green) domains of subunit A are labeled, and subunit B is colored in yellow. PLP is shown as spheres. The different conformations of K240 and H241 in apo-*Lb*TDC and holo-*Lb*TDC are shown in the box. Residue S440 from the other subunit is shown in magenta. The 2Fo-Fc map omitting PLP contoured at 2σ is shown (mesh). Hydrogen bonds are depicted as red dashed lines. Water molecules are shown as red spheres.

The overall structure of *Lb*TDC is shown in Fig. 1. *Lb*TDC is a homodimer, and the two subunits exist in an asymmetric form. Compared with the open conformation of the apo form of the human DDC structure^30^, the structure of apo-*Lb*TDC is in a closed conformation. Because there are no cysteines in *Lb*TDC, the two subunits are linked to each other by a hinge-like domain consisting of two long, parallel α-helixes. Similar to the structures of other Group II decarboxylases, each *Lb*TDC monomer is composed of three distinct domains^31^: the *N*-terminal (residues 7–105) domain, the PLP-binding (residues 106–462) domain, and the small (residues 463–618) domain. The *N*-terminal domain is the hinge-like domain that intertwines between the two subunits and contributes to the stability of the dimer structure. This *N*-terminal domain is connected to the PLP-binding domain by a long loop. The PLP-binding domain is composed of a typical seven stranded β-sheet surrounded by thirteen α-helixes, which form the dimer interface. The small (or C-terminal) domain contains a five-stranded β-sheet and five α-helixes. The active center is located in a shallow cavity at the interface between the two subunits of the dimer. Residues from both subunits are involved in cofactor binding^31^.

Tyrosine decarboxylase from *Methanococcus jannaschii* (*Mj*TDC) is the only TDC structure deposited in PDB under accession number 3F9T and was solved at 2.1 Å resolution. A comparison of the sequences of *Lb*TDC and *Mj*TDC reveals very different lengths. The structures of other Group II decarboxylases have been published, such as DDC from pig (1JS3, 2.25 Å), HDC (4E1O, 1.8 Å) and GAD (2OKJ, 2.3 Å) from humans. Although they exhibit relatively low amino acid sequence identities (<30%) with *Lb*TDC, their spatial structures were relatively conserved. All of them consist of three characterized domains and have similar active centers and conserved essential residues, such as the tyrosine in the flexible loop, which is vital for protonation.

In the electron density map, some of the amino acids in the loop region (from 415 to 431) are invisible, and could not be resolved due to substantial disorder. This flexible loop region is located near the substrate-binding site, and is conserved in other Group II decarboxylases. It has been shown that this loop might act as a solvent and substrate/product lid^32^. The conserved tyrosine residue (Y420) in the loop region is responsible for protonating C_α_ in the transition state^32^,^33^,^34^,^35^. Y420A and Y420F mutants were constructed by site-directed mutagenesis. No measureable activity was detected, even in the presence of excess substrate, suggesting the vital role of Y420 in the decarboxylation activity of *Lb*TDC.

### PLP-binding site

*Lb*TDC is a PLP-dependent enzyme, and the PLP cofactor is covalently attached to the ε-amino group of K392 to form an internal aldimine via a Schiff-base interaction (Fig. 1). The binding between PLP and the enzyme is structurally and functionally conserved in most of the PLP-dependent enzymes. The protonated pyridine nitrogen of PLP forms a pair of salt bridges to the carboxyl group of D328. Moreover, the PLP pyridine ring is anchored by the methyl group of A330 and the imidazole ring of H241. The O3 atoms of the pyridine ring of PLP seem to interact with T298, K392 and two adjacent water molecules. The phosphate moiety of PLP is stabilized by a number of interactions with G158, S159, and D389 of one subunit and S440 of the other subunit.

### The role of H241

A comparison of the structure of apo-*Lb*TDC with the structure of holo-*Lb*TDC revealed that there are two opposite conformations of K240 and H241 (Fig. 1). In the apo-*Lb*TDC structure, the imidazole ring of H241 is anchored by two water molecules and is distant from the PLP binding pocket, and the side chain of K240 is placed in a position near the PLP binding pocket. However, in the holo-*Lb*TDC structure, the positions of K240 and H241 are rotated to the opposite orientation. Residues K240 and H241 form a short flexible loop. In chain B of holo-*Lb*TDC, a conformational shift of this short flexible loop was captured, in contrast to the apo-*Lb*TDC structure. The imidazole ring of H241 swings to the pyridine ring of PLP. Additionally, through a structural alignment with drDDC and pig DDC, a conserved histidine (H192) was also identified, which has been reported to interact with the pyridine ring of PLP. This interesting dynamic change in conformation suggests that K240 and H241 play important roles in establishing the conformation required for the binding and stabilization of cofactor and substrate.

**Figure 2:**
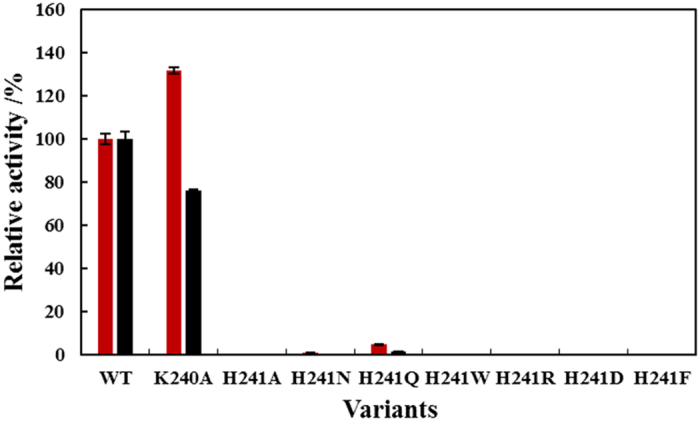
Mutagenesis of K240 and H241. (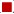) tyrosine, (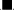) DOPA. The activities of the WT enzyme toward tyrosine and DOPA are set to 100%.

To investigate the role of K240 and H241 in *Lb*TDC, semi-saturation mutagenesis of H241 (H241A, H241N, H241Q, H241W, H241R, H241D, and H241F) was performed, and the K240A mutant was created (Fig. 2). All variants were purified for the activity and kinetic analyses, and the mutations had no significant effect on the expression levels and solubility of *Lb*TDC. Compared with the wild type (WT) protein, K240A exhibits an increased *k*_cat_ toward tyrosine and a decreased *k*_cat_ toward DOPA. Additionally, higher *K*_m_ values were observed for both tyrosine and DOPA using the K240A mutant (Table 2). As a result, the *k*_cat_/*K*_m_ of K240A toward both tyrosine and DOPA was also decreased, indicating that mutation of lysine-240 to alanine might affect the affinity of enzyme and substrates by altering the conformation of adjacent amino acids, particularly H241. Most of the H241 mutants completely lost their decarboxylase activity, but H241N and H241Q retained low activities toward tyrosine and DOPA. We failed to determine the kinetic parameters of H241N and H241Q, because their *K*_m_ values were too high to be determined within the solubility of tyrosine (5.5 mM). Nevertheless, it can still be concluded that the substrate affinity of the H241 variants is significantly decreased, based on the *K*_m_ of the WT enzyme toward tyrosine (0.58 mM). We supposed that the mutations of K240 and H241 influenced the apo-holo transition and led to weak PLP binding and the loss of decarboxylase activity. K240 and H241 are key residues required for the stabilization of substrate and cofactor. A sequence alignment of *Lb*TDC and other Group II decarboxylase reveals that His241 is highly conserved^36^. In the crystal structures of drDDC and pig DDC, the corresponding histidine (H192 in drDDC and pig DDC) interacts with the PLP pyridine ring and the inhibitor (cabiDOPA), respectively^32^,^37^. The residues corresponding to position 240 in most Group II decarboxylases are mainly alanine, instead of the lysine observed in *Lb*TDC. In this study, the K240A mutant displayed a decreased *k*_cat_/*K*_m_, suggesting a beneficial role of K240 in *Lb*TDC.

### Site-directed mutagenesis around the active site

**Figure 3:**
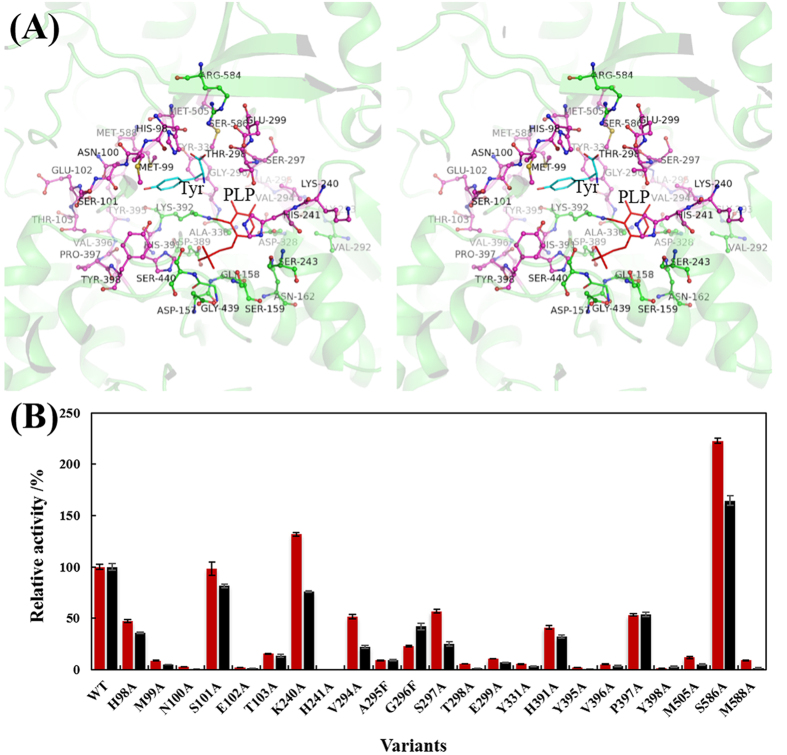
(**A**) Stereo views of the catalytic center of *Lb*TDC. The residues that were mutated in this study are shown in magenta. (**B**) Relative activities of *Lb*TDC and its variants toward tyrosine and DOPA. PLP and the docked substrate tyrosine are shown in red and cyan, respectively. (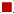) tyrosine, (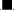) DOPA. The activities of the WT enzyme toward tyrosine and DOPA are set to 100%.

Despite the various conditions used to co-crystallize *Lb*TDC with tyrosine or inhibitors (tyrosine methyl ester and tyrosine amide), crystals of the complex with the substrate or inhibitors were not obtained. To further investigate the role of the residues around the catalytic center, tyrosine was docked into the holo-*Lb*TDC structure. Due to its critical role in Parkinson disease, a number of site-directed mutagenesis studies have been conducted on DDC to understand its catalytic mechanism. The K303A mutant showed a 1,500-fold decrease in decarboxylation activity. The kinetic analysis reveals that Lys303 plays an irreplaceable role in the formation of the external aldimine, hydrolysis and product release^38^. The increased *K*_m_ values of the T82A and T82S mutants suggest that T82 is involved in substrate binding^36^. Mutation of residues D271, H192, H302, and N300 shows that these residues are not essential but can alter the nature of catalysis^39^. A number of residues within the loops around the putative substrate binding pocket (as illustrated in Fig. 3A) of *Lb*TDC were selected for the site-directed mutagenesis study. All of the residues were mutated to alanine (except for A295F and V296F) because it displays no polar character and less steric hindrance. All variants were purified, and the specific activities were analyzed (Fig. 3B).

Our results show that mutagenesis of the residues between 98 and 103, which are located in a loop region, led to decreased decarboxylase activity. The H98A and S101A variants retained approximately 40% and 80% of their activity, respectively, whereas the other mutants were almost completely inactive. Although mutations of residues 294–299 also resulted in decreased activity, a different substrate preference was noticed in variants, such as G296F, which displayed higher activity toward DOPA than tyrosine compared with WT. Therefore, residues 294–299 are considered important for substrate specificity. Future studies could analyze this region to obtain TDC variants with decarboxylase activity toward different amino acids.

**Figure 4:**
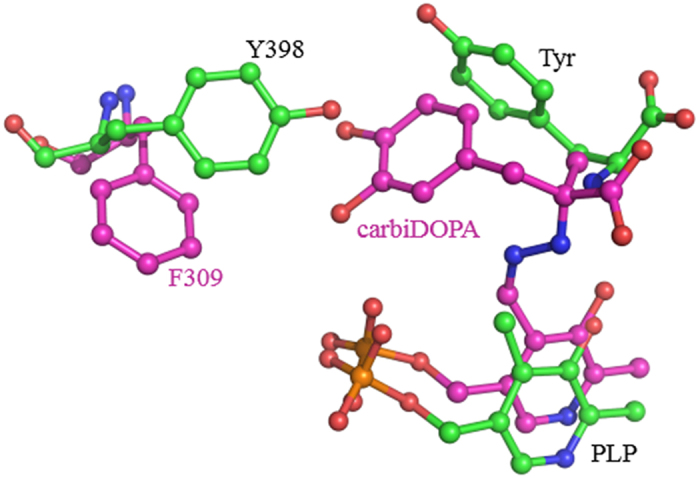
Superposition of the *Lb*TDC structure onto the pig DDC structure. Residues from pig DDC are colored in magenta, and those from *Lb*TDC are colored in green.

Residues 395–398, which are close to the substrate, and Y331 and H391 were also mutated to alanine. H391A and P397A showed approximately 40% and 50% of the activity of the WT, respectively, whereas the others displayed extremely low activities (<5%). No obvious change in substrate preference was observed in the variants. Further saturation mutagenesis would be necessary to gain deeper insights into the role of this loop. The specific activity of the Y398A mutant was significantly decreased toward both tyrosine and DOPA. Superposition of the pig DDC structure onto the *Lb*TDC structure reveals that the Tyr398 position in *Lb*TDC is a phenylalanine in pig DDC (Fig. 4). Hence, Y398F and Y398A mutants were further characterized to clarify the role of Y398. The *K*_m_ values of the Y398A variant toward tyrosine and DOPA are 5- and 2-fold higher than that of the WT enzyme, respectively. For the Y398F variant, the *K*_m_ toward tyrosine is increased more than 4-fold, whereas its *K*_m_ toward DOPA is slightly reduced by 13%. The *k*_cat_ values of Y398F and Y398A toward both substrates are significantly decreased (Table 2). The results indicate that Y398 is important for the binding to the amino acid substrate.

**Figure 5:**
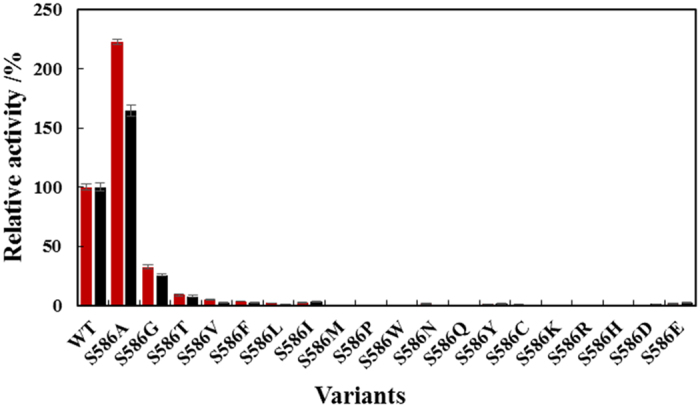
Saturation mutagenesis of S586. (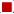) tyrosine, (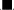) DOPA. The activities of the WT enzyme toward tyrosine and DOPA of the WT are set to 100%.

**Figure 6:**
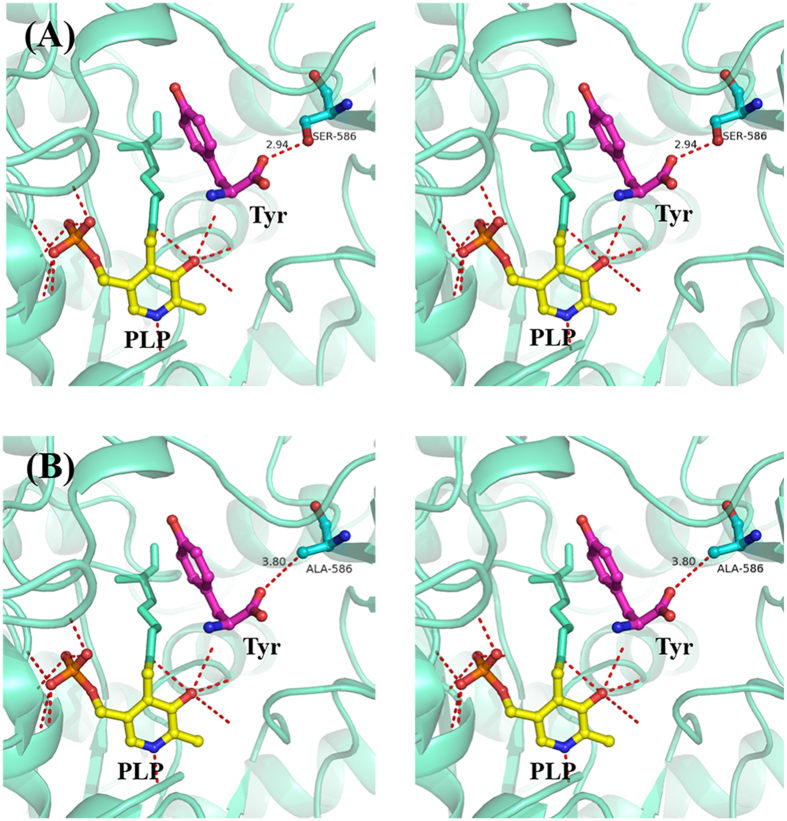
Stereo views of substrate binding of the WT and S586A. (**A**) S586 and (**B**) A586. PLP and the docked substrate tyrosine are shown in yellow and magenta, respectively. Residue interactions are depicted as red dashed lines.

The M505A, S586A, and M588A variants were also constructed and purified. Interestingly, S586A displayed an over 2-fold improvement in activity compared with the WT enzyme (96.0 ± 2.0 U·mg^−1^ versus 43.1 ± 1.0 U·mg^−1^), representing the highest activity among all variants tested. The kinetic parameters of S586A were also determined. The *k*_cat_ and *K*_m_ values of S586A toward tyrosine and DOPA are 250.9 ± 4.2 s^−1^ and 0.42 ± 0.03 mM and 126 ± 3.48 s^−1^ and 0.69 ± 0.07 mM, respectively. Its *k*_cat_/*K*_m_ values toward tyrosine and DOPA are 600 and 181 s^−1^·mM^−1^, respectively, which are 2.78- and 1.79-fold higher than the WT enzyme. A sequence alignment with other Group II decarboxylases indicates that the residues at 586 are not conserved. Considering the beneficial effect of the alanine mutation at S586, saturation mutagenesis was performed to elucidate its specific mechanisms. In addition to S586A, S586G and S586T retained more than 10% activity toward tyrosine, whereas the other mutants showed less than 5% or undetectable activity toward tyrosine or DOPA (Fig. 5). These results suggest that S586 is a critical residue for substrate binding. Based on the activities of S586A, S586G, and S586T compared with WT, it is speculated that there are specific requirements regarding the steric hindrance and hydrophobicity of residue 586. Only amino acids with small or similar side chains to serine at position 586 retained decarboxylase activity, suggesting that reduced steric hindrance is advantageous. Additionally, the docking result indicates that the distance between S586 and tyrosine is only 2.9 Å (Fig. 6). Importantly, hydrophobicity is also attributed to the higher hydropathy index of alanine (2.5), which is higher than glycine (−0.4), serine (−0.8), and threonine (−0.7)^40^, and variant S586A displayed the highest decarboxylase activity. The higher hydrophobicity at position 586 might conduce to enhanced binding energy for tyrosine.

In summary, we determined the crystal structure of *Lb*TDC in complex with PLP at 1.9 Å. Semi-saturation mutagenesis on the conformationally flexible H241 residue shows that only H241N and H241Q retained low activity, indicating that H241 is vital for the conformation required to bind PLP and tyrosine. Alanine scanning mutagenesis revealed several residues that influence activity and specificity, including G296, Y398, and S586. Saturation mutagenesis was performed on S586. The *k*_cat_/*K*_m_ values of the S586A variant toward tyrosine and DOPA are 600 and 181 s^−1^·mM^−1^, which are 2.78- and 1.79-fold higher than the WT enzyme. Decreased steric hindrance and increased hydrophobicity are possible reasons for the improved catalytic efficiency of S586A. This study provided a structural basis for the further engineering and application of TDC in the biocatalytic synthesis of tyramine.

## Additional Information

**How to cite this article**: Zhu, H. *et al.* Crystal structure of tyrosine decarboxylase and identification of key residues involved in conformational swing and substrate binding. *Sci. Rep.* **6**, 27779; doi: 10.1038/srep27779 (2016).

## Supplementary Material

Supplementary Information

## Figures and Tables

**Table 1:** Data collection and refinement statistics of the TDC structure alone and in complex with PLP.

	5HSI (without PLP)	5HSJ (complex with PLP)
Data collection statistics		
Cell parameters		
a, b, c (Å)	61.8, 126.2, 84.8	61.9, 126.8, 82.9
α, β, γ (°)	90.0, 113.4, 90.0	90.0, 109.7, 90.0
Resolution (Å)	50–1.73 (1.79–1.73)	50–1.90 (1.97–1.90)
Wavelength (Å)	1.54178	0.97908
Space group	P2_1_	P2_1_
Total reflections	483537	488739
Completeness (%)	90.7 (89.2)	99.7 (99.9)
R_merge_ (%)^b^	5.1 (23.8)	11.9 (77.0)
Redundancy	4.3 (4.3)	5.2 (5.2)
I/error	12.4 (3.8)	10.0 (3.0)
Refinement statistics		
R_work_/R_free_ (%)	15.52/19.05	17.13/21.28
Average B factor (Å^2^)		
Protein	25.1	33.1
Ligand	23.8	32.2
Solvent	34.3	37.2
R.M.S. deviations from ideal geometry		
Bond length (Å)	0.006	0.007
Bond angle (°)	1.074	1.041
Ramachandran plot		
Favored (%)	97.06	97.65
Allowed (%)	2.61	2.10
Outliers (%)	0.34	0.25
No. atoms		
No. of non-hydrogen atoms	10,743	10,144
No. of amino acid residues	9,541	9,542
No. of solvent molecules	1,201	569

^a^The values in parentheses represent the highest resolution shell. ^b^R_merge_ =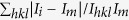, where *I*_i_ and *I*_m_ are the observed intensity and the mean intensity of related reflections, respectively. The values in parentheses indicate the highest resolution shell.

**Table 2:** Kinetic parameters of *Lb*TDC and the mutant proteins toward tyrosine and DOPA.

Substrate	Enzyme	Specific activity [U·mg^−1^]	*K*_m_ [mM]	*k*_cat_ [s^−1^]	*k*_cat_/*K*_m_ [s^−1^·mM^−1^]
Tyrosine					
	WT	43.1 ± 1.0	0.6 ± 0.1	124.8 ± 1.5	216.0
	K240A	56.8 ± 0.7	1.6 ± 0.2	224.5 ± 11.9	139.4
	H241N	0.2 ± 0.1	ND^a^	ND	ND
	H241Q	1.9 ± 0.1	ND	ND	ND
	S586A	96.0 ± 2.0	0.4 ± 0.1	250.9 ± 4.2	600.4
	Y398A	0.6 ± 0.1	3.0 ± 0.5	9.8 ± 0.8	3.4
	Y398F	4.2 ± 0.2	2.4 ± 0.5	31.9 ± 3.4	13.5
DOPA					
	WT	26.5 ± 0.9	0.8 ± 0.1	76.5 ± 3.0	101.1
	K240A	20.1 ± 0.2	1.1 ± 0.2	64.7 ± 3.0	58.3
	H241N	n.d.^b^	ND	ND	ND
	H241Q	0.3 ± 0.1	ND	ND	ND
	S586A	43.7 ± 1.5	0.7 ± 0.1	126 ± 3.5	181.3
	Y398A	0.8 ± 0.2	1.7 ± 0.2	2.5 ± 0.2	1.5
	Y398F	3.2 ± 0.4	0.7 ± 0.1	12.3 ± 0.3	18.4

^a^ND, not determined.

^b^n. d.: no activity was detected.
