# Supplementary material for: Crystal structure of tyrosine decarboxylase and identification of key residues involved in conformational swing and substrate binding
Source: Sci Rep. 2016 Jun 13;6:27779. doi: 10.1038/srep27779 (PMC4904194; doi:10.1038/srep27779)
Supplement: Supplementary Information [file srep27779-s1.pdf]

Supplementary Information

Crystal structure of tyrosine decarboxylase and identification of key residues involved in conformational swing and substrate binding

Haixia Zhu[1,2], Guochao Xu[1], Kai Zhang[1], Xudong Kong[3], Ruizhi Han[1], Jiahai Zhou[2,]* and Ye Ni[1,]*

[1]The Key Laboratory of Industrial Biotechnology, Ministry of Education, School of Biotechnology, Jiangnan University, Wuxi 214122, Jiangsu, Peoples R China

[2] Shanghai Institute of Organic Chemistry, State Key Laboratory of Bioorganic & Natural Products Chemistry, Chinese Academy of Sciences, Shanghai 200032, Peoples R China

[3] State Key Laboratory of Bioreactor Engineering, School of Biotechnology, East China University of Science and Technology, Shanghai 200237, Peoples R. China

*Corresponding authors: **Prof. Y. Ni**, *yni@jiangnan.edu.cn*

Prof. J. H. Zhou, *jiahai@mail.sioc.ac.cn*

Table S1 Primers used in this study.

H98A-F	TGGGGTGCAATGAATTCAGAG	S586G-F	CGCGCTGGTGTTATGACGCCG
H98A-R	ATTCATTGCACCCCAATAACG	S586G-R	CATAACACCAGCGCGAATGAT
M99A-F	GGTCACGCAAATTCAGAGACC	S586V-F	CGCGCTGTTGTTATGACGCCG
M99A-R	TGAATTTGCGTGACCCCAATA	S586V-R	CATAACAACAGCGCGAATGAT
N100A-F	CACATGGCATCAGAGACCCTA	S586L-F	CGCGCTCTGGTTATGACGCCG
N100A-R	CTCTGATGCCATGTGACCCCA	S586L-R	CATAACCAGAGCGCGAATGAT
S101A-F	ATGAATGCAGAGACCCTAATG	S586I-F	CGCGCTATTGTTATGACGCCG
S101A-R	GGTCTCTGCATTCATGTGACC	S586I-R	CATAACAATAGCGCGAATGAT
E102A-F	AATTCAGCAACCCTAATGCCT	S586M-F	CGCGCTATGGTTATGACGCCG
E102A-R	TAGGGTTGCTGAATTCATGTG	S586M-R	CATAACCATAGCGCGAATGAT
T103A-F	TCAGAGGCACTAATGCCTGCT	S586P-F	CGCGCTCCGGTTATGACGCCG
T103A-R	CATTAGTGCCTCTGAATTCAT	S586P-R	CATAACCGGAGCGCGAATGAT
K240A-F	CAAACGGCACATTATTCTTGG	S586F-F	CGCGCTTTTGTTATGACGCCG
K240A-R	ATAATGTGCCGTTTGTGGTAC	S586F-R	CATAACAAAAGCGCGAATGAT
H241A-F	ACGAAGGCATATTCTTGGATC	S586W-F	CGCGCTTGGGTTATGACGCCG
H241A-R	AGAATATGCCTTCGTTTGTGG	S586W-R	CATAACCCAAGCGCGAATGAT
V294A-F	GTTGGTGCAGCCGGATCAACT	S586C-F	CGCGCTTGTGTTATGACGCCG
V294A-R	TCCGGCTGCACCAACCACGCC	S586C-R	CATAACACAAGCGCGAATGAT
A295F-F	GGTGTGTTTGGATCAACTGAA	S586T-F	CGCGCTACCGTTATGACGCCG
A295F-R	TGATCCAAACACACCAACCAC	S586T-R	CATAACGGTAGCGCGAATGAT
G296F-F	GTGGCCTTTTCAACTGAAGAA	S586N-F	CGCGCTAATGTTATGACGCCG
G296F-R	AGTTGAAAAGGCCACACCAAC	S586N-R	CATAACATTAGCGCGAATGAT
S297A-F	GCCGGAGCAACTGAAGAAGGT	S586Q-F	CGCGCTCAGGTTATGACGCCG
S297A-R	TTCAGTTGCTCCGGCCACACC	S586Q-R	CATAACCTGAGCGCGAATGAT
T298A-F	GGATCAGCAGAAGAAGGTGCC	S586Y-F	CGCGCTTATGTTATGACGCCG
T298A-R	TTCTTCTGCTGATCCGGCCAC	S586Y-R	CATAACATAAGCGCGAATGAT
E299A-F	TCAACTGCAGAAGGTGCCGTT	S586K-F	CGCGCTAAAGTTATGACGCCG
E299A-R	ACCTTCTGCAGTTGATCCGGC	S586K-R	CATAACTTTAGCGCGAATGAT
Y331A-F	GCTGCAGCAGGTGGATATGCT	S586R-F	CGCGCTCGTGTTATGACGCCG
Y331A-R	TCCACCTGCTGCAGCATCTAC	S586R-R	CATAACACGAGCGCGAATGAT
H391A-F	GATCCCGCAAAGATGGGATAT	S586H-F	CGCGCTCATGTTATGACGCCG
H391A-R	CATCTTTGCGGGATCAATTGT	S586H-R	CATAACATGAGCGCGAATGAT
G394F-F	AAGATGTTTTATGTACCATAC	S586D-F	CGCGCTGATGTTATGACGCCG
G394F-R	TACATAAAACATCTTATGGGG	S586D-R	CATAACATCAGCGCGAATGAT

Y395A-F	ATGGGAGCAGTACCATACTCG	S586E-F	CGCGCTGAAGTTATGACGCCG
Y395A-R	TGGTACTGCTCCCATCTTATG	S586E-R	CATAACTTCAGCGCGAATGAT
V396A-F	GGATATGCACCATACTCGGCT	H241R-F	ACGAAGCGTTATTCTTGGATG
V396A-R	GTATGGTGCATATCCCATCTT	H241R-R	AGAATAACGCTTCGTTTGTGG
P397A-F	TATGTAGCATACTCGGCTGGG	H241w-F	ACGAAGTGGTATTCTTGGATG
P397A-R	CGAGTATGCTACATATCCCAT	H241w-R	AGAATACCACTTCGTTTGTGG
Y398A-F	GTACCAGCATCGGCTGGGGGC	H241L-F	ACGAAGCTGTATTCTTGGATG
Y398A-R	AGCCGATGCTGGTACATATCC	H241L-R	AGAATACAGCTTCGTTTGTGG
M505A-F	TTCAATGCAGTTGACTATGTT	H241S-F	ACGAAGAGCTATTCTTGGATG
M505A-R	GTCAACTGCATTGAAGTCAGG	H241S-R	AGAATAGCTCTTCGTTTGTGG
S586A-F	CGCGCTGCAGTTATGACGCCG	H241D-F	ACGAAGGATTATTCTTGGATG
S586A-R	CATAACTGCAGCGCGAATGAT	H241D-R	AGAATAATCCTTCGTTTGTGG
M588A-F	TCGGTTGCAACGCCGTATATG	H241F-F	ACGAAGTTTTATTCTTGGATG
M588A-R	CGGCGTTGCAACCGAAGCGCG	H241F-R	AGAATAAAACTTCGTTTGTGG
Y398F-F	GTACCATTTTCGGCTGGGGGC	H241N-F	ACGAAGAATTATTCTTGGATG
Y398F-R	AGCCGAAAATGGTACATATCC	H241N-R	AGAATAATTCTTCGTTTGTGG

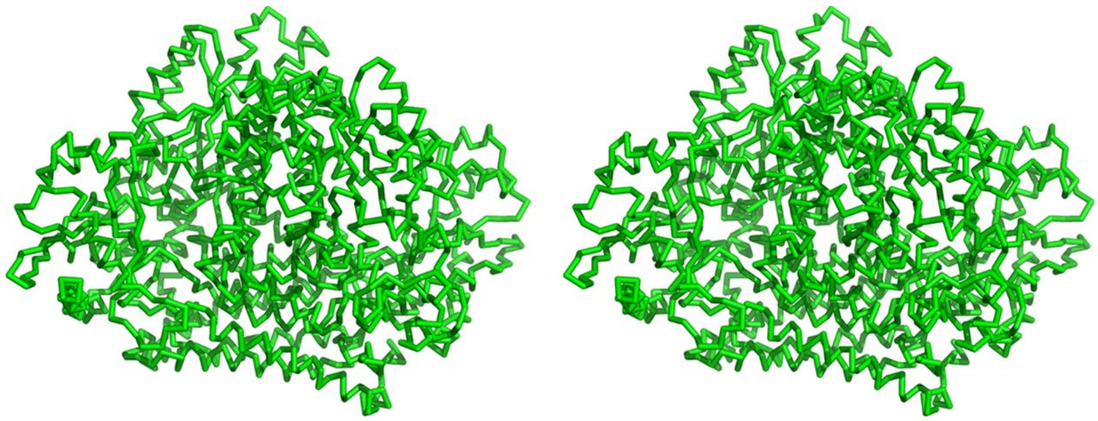

Figure S1 Stereo image of the entire structure of *Lb*TDC.
